# Truth is relative; Client report versus provider report, a post-modern analysis of data from a trial in the private retail medicine sector

**DOI:** 10.21203/rs.3.rs-5005686/v1

**Published:** 2024-10-21

**Authors:** Tabitha Chepkwony, Mark E. Amunga, Emmah Kimachas, Joseph Kipkoech, Emily Robie, Aggrey Wekesa, David Arthur, Elizabeth L. Turner, John A. Gallis, Lucy Abel, George Ambani, Theodoor Visser, Meley Woldeghebriel, Sameen Babur, Aaron Woolsey, Diana Menya, Jeremiah Laktabai, Wendy P. O’Meara

**Affiliations:** AMPATH; AMPATH; AMPATH; AMPATH; Duke University NC; Duke Global Inc; Duke University NC; Duke University NC; Duke University NC; AMPATH; AMPATH; Clinton Health Access Initiative (CHAI); Clinton Health Access Initiative (CHAI); Clinton Health Access Initiative (CHAI); Clinton Health Access Initiative (CHAI); Moi University; Moi University; Duke University NC

**Keywords:** ACT, Malaria, Private retail sector, mRDT, Subsidies, Subsidy, Exit interviews

## Abstract

In malaria-endemic countries, private retail outlets are a major source of antimalarials for individuals experiencing an acute febrile illness. However, there remains a challenge in how the decision to dispense the drugs is made. The lack of malaria diagnostic tools in the retail sector leads to a presumptive approach to diagnosis and overuse of ACTs. The TESTsmART study trained retail outlet attendants to perform malaria rapid diagnostic tests (mRDTs) in conjunction with a mobile application to capture testing and drug dispensing data. Concurrently, febrile clients were randomly selected for exit interviews outside the outlet, and analogous information about testing and drug purchasing was recorded based on self-report. A small subset of clients enrolled in exit interviews were also asked to participate in exit *Plasmodium falciparum* mRDT testing to confirm the accuracy of mRDTs in the outlet and to estimate malaria positivity amongst untested clients.

In this sub-study, comparison of these two concurrent data sources showed the testing rate for eligible participants was slightly lower in the exit interview (42.8%, 2436/5695) than in the app (51.1%, 24,446/49,804). We noted important differences in the experiences of testing and adherence reported by outlets compared to clients; 11.0% of clients had positive mRDT reported in the app (and validated by photo review) compared to 35.3% from exit interviews. Outlets reported that 97% of test-positive clients received a first-line Artemether Combination Therapy (ACT), but only 77% of clients who reported a positive test also reported receiving the first-line ACT in the exit interview. For test-negative clients, 35% received an ACT based on outlet reports compared to 25% by exit interviews. Among 109 clients randomly selected for re-test at exit interview, nearly two-thirds of those who reported a positive test from the outlet had a negative mRDT (64.3%, 9/14) when retested. Contrasting outcomes reported by the provider and the client highlight barriers to improving testing and adherence for malaria as well as challenges for monitoring case management in the retail sector. These include accurate communication of results to the client, poor confidence in a negative result, and reluctance to withhold antimalarials from test-negative clients.

## INTRODUCTION

Malaria continues to exact a heavy toll globally, with 247 million cases reported in 2021, the majority of which were in Africa (1). Infection with malaria parasites can cause a range of symptoms, from asymptomatic or mild to severe disease or even death. Symptoms are non-specific, and distinguishing fevers from malaria from those due to other causes is impossible based on clinical presentation. To improve malaria case management and appropriate use of first-line antimalarials, the WHO recommends that all suspected malaria cases be confirmed parasitologically, followed by treatment of confirmed cases with artemisinin combination therapy (ACT) (2).

In malaria-endemic countries, private retail outlets are a major source of ACTs for those experiencing a febrile illness. However, there remains a challenge in how the decision to dispense the drugs is made. Due to the lack of malaria diagnostic tools in the retail sector (3, 4), most clients with suspected malaria are treated without confirmatory testing, resulting in poor targeting of ACTs and inappropriate treatment of undiagnosed illnesses. Although integrating parasitological diagnosis into the retail sector may seem strategic for reducing the overuse of antimalarials, studies designed to measure the impact of point-of-care malaria testing in retail outlets have shown inconsistent impact on ACT dispensing and highlighted challenges with aligning private sector services with public health goals such as Test before Treat (5–7). Several countries have approved using malaria rapid diagnostic tests (mRDTs) in retail outlets (7). However, it is unclear what impact these policies have had on malaria case management due to the private sector’s lack of reporting (8).

Using data from the TESTsmART trial, a cluster randomized controlled trial conducted in 39 retail medicine outlets in western Kenya between October 2020 and September 2022 was designed to improve parasitological diagnosis of suspected malaria cases and targeting ACTs to those with confirmed infection. Throughout the 24- month study period, retail outlets captured information about individuals who sought treatment for malaria-like illnesses through use of a mobile app. Specifically, they captured whether or not those treatment-seeking individuals were tested for malaria, the test results, and ultimately the medicines purchased. Concurrently, the study also interviewed clients exiting retail outlets on random days to record information about malaria testing, test results, and medicines purchased during their visit.

In this analysis we look at data from these two sources – one reported by outlet staff and the other from the client self-report at the exit interview – to compare the testing and treatment experiences from the two sides. Part of the goal was to find out how reliable outlet-reported data was compared with the exit interview data and whether the information can be used to upscale mRDT testing in the private retail outlets. We also identify similarities in the two populations by age, gender, and test uptake and highlight differences in test results and drugs dispensed as reported by the two sources of information. These observations are supplemented with exit testing on a subsample of participants, further highlighting discrepancies in diagnostic outcomes.

## METHODS

The trial aimed to evaluate the degree to which incentives directed at the provider or client affect the purchasing behavior of all suspected malaria cases seeking treatment in enrolled retail medicine outlets with respect to their willingness to undergo malaria diagnostic testing and to purchase appropriate treatments. In this paper, we report findings from a comparison of two data sources of malaria rapid diagnostic testing and treatment targeting the same participants.

### Study Site

The study was conducted in Bungoma and Trans Nzoia counties, rural communities in a malaria-endemic region of western Kenya where approximately 30% of fevers are due to malaria (12) and malaria rapid diagnostic testing is not currently available in the private drug retail sector. The study took place in private retail outlets at the selected sites.

### Study Design

Data from this paper came from a three-arm cluster-randomized trial that included 39 registered medicine retail outlets selected from a complete sampling frame of all eligible outlets and which were randomized 1:1:1 to one of three study arms: control, client-directed incentives or a combined intervention with both client-directed and provider-directed incentives. Outlets in all three arms had access to malaria Rapid Diagnostics Test kits (mRDTs) at a wholesale price. This enabled the outlets to sell them to the clients for a retail price of 40 Kenyan shillings (approximately USD $0.40). In all the arms, outlet owners/attendants were trained on malaria testing using mRDT and the TESTsmART mobile reporting app. Clients who did not wish to purchase an mRDT were free to conduct their transactions as desired. Full details of the trial can be found at (9, 10). Eligible clients were those with a fever or history of fever in the last 48 hours or who suspected that they had malaria and who were older than one year, information on clients who were unwell and had sent someone else to purchase drugs at the outlet was also captured in the app. In the current analysis, we have aggregated client-level data across the arms and ignored the intervention assignment due to the absence of an intervention effect on the main outcomes of testing and adherence to test results (11).

Client-level data were collected in three ways − 1) outlet attendants entered information about clients seeking care for acute, malaria-like illness into the TESTsmART app, 2) on random days of the week, field researchers interviewed clients exiting the outlet about their illness, testing and purchases made at the outlet, and 3) exit malaria testing was conducted on a small random sample of exit interview clients as a Quality Control (QC) measure. In this analysis, we compare client-level data from these three sources.

### Data Sources

#### The Mobile Application

Independent of the study outcomes data collection, we deployed a mobile app in each of the 39 outlets and which was designed explicitly for the TESTsmART study in order to capture individual client encounters. The mobile app was built on an Android platform with a cloud-syncing function and consisted of three main modules: Customer registration (age, gender, symptoms), Diagnosis, and Treatment. The diagnosis module provided camera functionality enabling image capture of mRDT cassettes, which were subsequently synced to a server. The treatment module captured the medications that were dispensed. All data was manually entered by the outlet attendant. Outlet attendants were trained on mRDT performance procedures and mobile app use. Once data were uploaded, a trained laboratory scientist or clinical officer reviewed mRDT images to check for mRDT test quality and accuracy of reported results and provided feedback for improvement. ACT subsidies for the two study arms offering client-directed incentives were prescribed through the app when clients tested positive.

Data reported through the app were primarily used to track mRDT and ACT sales in real-time and was regularly reviewed to track the proportion of positive tests, the volume of mRDTs used, and the quality of uploaded mRDT photos. This routine monitoring helped to detect potential problems (i.e., providers who had unusually high- or low-test positivity rates and problems with mRDT interpretation). Problems detected then triggered support supervision and/or additional on-the-job training to ensure compliance and quality of diagnosis.

#### Exit Interviews

This data source was used to evaluate the main study outcomes reported in (9). Exit interviews were conducted on random days each month at each participating outlet with data entered directly into Redcap. Clients leaving the private retail outlet were asked to participate in a brief survey. If the individual with symptoms was less than 18 years, the parent was consented and interviewed about the child. Clients with severe illness requiring immediate referral, had taken an antimalarial in the last 7 days, were purchasing drugs on behalf of someone not present, and those below 18 years of age without a parent or legal guardian present or otherwise unable to consent were not eligible and hence not interviewed. Those who met the inclusion criteria then verbally consented before being interviewed with questions about their current illness and their decisions regarding testing and medicines purchased. The interview information was captured on Redcap. The exit interview was conducted in one session and lasted approximately 15–20 minutes.

#### Exit Testing

To confirm the accuracy of malaria diagnostic testing in participating outlets and to measure how many fever clients missed getting an appropriate drug when treated over the counter without testing, we implemented random exit testing in a subsample of exit interview participants. During the final two months of the 24-month exit interview data collection period, two exit interview participants per outlet per day were offered a free of charge mRDT from a trained research assistant after they completed their transaction in the outlet. Some of these participants had already tested in the outlet. The test results of the exit test were recorded along with the participant’s study ID using a paper log and entered into an electronic log. If their test results at exit showed that they had malaria but did not purchase an antimalarial at the outlet, the study provided the appropriate dose of a government-recommended first-line antimalarial (Artemether-Lumefantrine). Participants provided written consent to participate in the exit testing subsample. Assent was sought from children between 8–17years of age.

#### Data Analysis

When analyzing these data, we note that individual encounter-level data could not be linked between the mobile app and exit interview since no personal identifying information was captured in either tool. Therefore, the analysis focused on differences in outcomes at the population-level rather than at the encounter or client level. However, the target population for data collection via the mobile app and the exit interview survey was designed to be the same, namely individuals experiencing malaria-like symptoms. In practice, the study team did not control which encounters the outlet attendant chose to enter in the app, and therefore the outlets may have exhibited some bias in prioritizing some entries over others. However, all mRDT results were accompanied by a photograph of the test which allowed the study team to verify all mRDT results recorded in the app. Analysis of app data is restricted to data from febrile individuals who were present at the retail outlet so as to mimic eligibility for the exit interview data source. Data on testing and medication purchasing are summarized as proportions and counts and reported by data source and by key demographic variables (gender and age group). All data analysis was performed using R software (version 4.3.2).

## RESULTS

### Analytic sample selection

From October 2020 - September 2022, the retail outlets captured 54,255 client encounters through the TESTsmART app (Table 1). The majority of these observations (91.8%) were eligible for inclusion in our current analysis because the client experiencing the febrile illness was present at the time of purchase. During the same 24-month study time frame, exit interviewers at participating retail outlets screened 11,783 clients, of whom 5,695 (48.3%) met eligibility criteria. Of the 5,831 who did not meet eligibility criteria, the major reason (61.5%, 3578/5831) was that the febrile individual was not present at the outlet, a much higher proportion than reported in the app. The percentage of those eligible who consented was 95.7% (5695/5952). Of those eligible, 5,695 (48.3%) consented to participate in the study.

### Analytic sample demographics

Among eligible encounters, the gender distribution was fairly even (49.5% male), and children under 18 were the most highly represented age group (35.2%), followed by 26–39-year-olds (27.1%), and 18–25-year-olds (20.0%). Of those eligible and who consented to the exit interview, a slightly larger proportion of exit interview participants was male (53.0%) compared to the percentage reported in the app (49.5%). Though children under 18 were again the most commonly observed clients (30.7%), 40–59-year-olds were the next highest-represented age group in the exit interviews (26.4%), followed by 26–39-year-olds (23.3%).

### Testing

Overall, 51.1% (n = 25446/49804) of eligible clients recorded in the app were tested by mRDT at the outlet compared to 42.8% (n = 2436/5695) of clients interviewed when exiting (Table 2). According to the data from the app, the difference between males and females in the proportion tested was small, just 4 percentage points. However, the exit interviews documented a difference of 10 percentage points in testing rates between males and females, with higher rates reported in females. Children had the highest testing rates, with similar proportions reported in both data sources. In older age groups, the difference between the two data sources widened. The exit interview data showed a much lower proportion of males than females being tested in older age groups.

### Test positivity

The self-reported test positivity rate was higher in the exit interview (35.1%) than in the outlet app (11.0%). There were no apparent differences in test positivity by gender in either data source. Children aged 1–17 years had the highest rate of positive mRDTs (42.0%) in exit interviews, while in the outlet app, test positivity peaked in adults between 18–25 years (13.1%) (Table 3). The absolute difference in test positivity between the two data sources was striking within each gender and across all age groups.

### Purchasing behavior and test adherence

The majority of the clients purchased 1–2 medicines regardless of their mRDT test status across both exit interview and outlet app data. In both data sources, clients who tested positive purchased more medicines (Table 4). For example, among exit interview participants, 41.2% of mRDT-positive participants purchased 3–4 drugs, whereas only 25.9% of mRDT-negative clients purchased as many drugs. The difference is more striking in the app, where 53.2% of mRDT positive purchased 3–4 drugs compared to 25.4% of mRDT negative. Few clients purchased no medications, although the proportions in both data sources were highest for clients with negative mRDT (23.0% in exit interviews, 15.7% in the app).

As a first line ACT, artemether lumefantrine (AL) was the most preferred antimalarial drug for treatment across all categories compared to other ACTs and non-ACT antimalarials. The reported use of AL was much higher among the malaria-positive individuals in the outlet app data at 85.5% (n = 2383/2,788) compared to 53.6% (n = 451/842) on Exit Interview. The proportion who purchased any ACT (AL or Other ACT) was similar in untested and test-positive cases when compared within each data source. However, clients recorded in the app had overall higher ACT consumption than the exit interviews. For example, 91.1% of untested clients recorded in the app were dispensed an ACT (71.9% AL and 19.2% Other ACT) compared to 70.8% of untested clients in the exit interviews (58.4% AL and 12.4% other ACT). Non-ACT antimalarials were dispensed infrequently regardless of testing status but were slightly higher for clients who were not present at the outlet (12.9% in the outlet app). Interestingly, 35.3% of mRDT-positive clients recorded in the app and 28.4% of mRDT-positive clients in exit interview received an injection, a much higher percentage than among untested (3.8% exit interview, 1.1% outlet app) or mRDT-negative clients (5.6% exit interview, 0.8% outlet app) in either data source.

Purchase of antibiotics among all those tested was similar in the exit interviews (mRDT positive 44.5% and mRDT negative 42.4%) and the app data (mRDT positive 42.2% and mRDT negative 40.6%), regardless of test result. Antibiotic purchases were lower among untested clients in both data sources (24.6% in exit interview and 20.7% in app). Painkillers were commonly dispensed to all categories.

Between June and August 2022,145 clients exiting outlets were enrolled in an exit testing sub study (Table 5). Of those enrolled, 24.8% (36/145) had already been tested in the outlet. Of those who tested at the outlet and the exit interview, 69.4% of the results agreed (25/36). Out of 14 who tested positive at the outlet, 5 (35.7%) were also positive at the exit test, while 9 (64.3%) received a negative test at exit. Of the 22 clients who tested negative at the outlet, 20 (90.9%) had exit results that agreed with the outlet testing, while 2 (9.1%) had a positive result at exit. Among those who were not tested at the outlet, only 3% (n = 4/109) had a positive mRDT at exit.

## DISCUSSION

The private retail sector is a major source of antimalarial drugs; globally, more than 40% of clients seek antimalarials distributed through the private sector (13). The introduction of mRDTs into the private retail sector could improve the diagnosis of those seeking treatment in retail outlets, hence facilitating proper treatment and reducing inappropriate use of antimalarials (14). However, understafinding the role of mRDTs in case management in the private sector is hampered by a lack of routine surveillance integrated into private sector operations. To understand the opportunities and limitations of routine surveillance in this context, we deployed an app designed to capture individual-level information about clients seeking antimalarials or treatment for malaria-like illnesses. The app was rolled out in outlets that were trained to conduct mRDTs and had access to a wholesale supply of mRDTs as part of a larger trial of mRDTs and ACT subsidies in the retail sector. We compare data that represent two independent perspectives - the provider perspective as captured in the app used by the outlet attendants and the client perspective captured by exit interviews conducted over the course of 24 months. The aim was to highlight the similarities and differences in testing uptake and treatment outcomes across the two data sources.

We identified significant discrepancies in the proportion of positive tests reported by the outlets compared to the clients. Across all age groups, the clients reported threefold higher positive mRDT results than the outlet report. We cannot definitively state why this discrepancy occurred, however it seems likely that miscommunication between the outlet attendant and the client regarding test results was a key contributor. This is confirmed by exit testing results which shows that a significant number of clients who self-reported positive results had a negative mRDT result on a second test. The client’s self-reported test result may not match the result of the mRDT if the attendant doubts the mRDT result and shares the findings with the client in an ambiguous way such as ‘the test doesn’t show your malaria yet’ or ‘this test can’t detect all the different species of malaria’, both of which were mentioned by attendants to the study team as concerns about mRDT validity. Other reports have shown that in some cases the attendant may simply deliver inaccurate information, especially when the mRDT result contradicts their clinical impression (15), or there is (implicit or explicit) pressure from the client that may believe they have malaria. Indeed, if the attendant dispenses an antimalarial following a test, the client may interpret this action to mean the test was positive for malaria, regardless of the actual results (15). This finding is consistent with a previous study that documented very high test positivity rates in outlets and positive mRDT results communicated to (malaria-free) mystery shoppers, raising concerns about client-provider communication and alignment of testing with outlets’ business practices (15). Shelus et al (16) also noted that the private outlet attendants were particularly concerned about losing money from sales if mRDT results were negative. They were conflicted between recommending best practices and losing business and wondered whether the public would consider them legitimate sources of malaria testing. It should be noted that this is not exclusively a private-sector problem; assignment of a diagnosis of malaria following a negative test result, and subsequent misinterpretation by the client, has been documented in public health facilities as well (17, 18)

The outlets and the clients also differed in their reporting of the types of medicines dispensed. The largest discrepancies were seen in ACTs dispensed to all categories of clients, which was substantially higher in the mobile app. Overall, more than 90% of clients were reported to have been dispensed an ACT by outlet staff, which was 20 percentage points higher than clients’ reports. The two sources of data did find similarly low use of non-ACT antimalarials, those not recommended by the Kenya Ministry of Health. However, they concurred on the frequent administration of injections, despite the fact that injections are not permitted in retail outlets. Outlet staff reported that a third of clients with a positive mRDT received an injection, and this was confirmed by the exit interview reports. This was also seen among registered and unregistered outlets in western Uganda and could be attributed to client or outlet attendant preferences (19). Most untested clients were given ACT, but fewer malaria-negative clients were given ACT, indicating the test likely influenced ACT dispensing. This finding was confirmed in the exit interview data. Dispensing ACTs to malaria-negative clients could be attributed to lack of alternative options in certain clinical findings by the provider (20).

Both data sources described patterns of dispensing non-malaria medications by testing status. Consumption of antibiotics was similar across both data sources. Notably, the purchase of antibiotics was twice as high for individuals with a test than for untested clients, regardless of the test result. It is interesting to note that mRDT testing did lead to lower ACT use among test-negative clients. However, contrary to our expectation, a positive test did not reduce presumptive antibiotic use. Painkillers were commonly dispensed to all clients. Therefore, the mRDT result does not appear to influence the decision of outlet staff on prescribing antibiotics and analgesics, similar to findings from a study in the public health sector in Mali (17).

Previous work on the use of exit interviews in the formal public health sector demonstrated good agreement between clinical records and exit interviews for specific actions such as testing or drug dispensing. However, there is poor agreement with more subjective practices such as dosing information or advice (21). This is consistent with our finding that understanding or interpretation of the outcome of the test is highly discordant, but testing rates and drug dispensing broadly agree between the two perspectives. In a study in Uganda where mRDTs were also deployed in retail outlets, results from household interviews and outlet reports (22, 23) show a large discrepancy in the prescription of ACTs to mRDT-negative clients. More than thirty percent of clients with a negative test reported receiving an ACT compared to 1.5% reported by the outlet attendants. Although the discrepancy we observe is related to dispensing ACT to mRDT-positive clients, both comparisons show the outlet is significantly more likely to report the ‘correct’ or desired behavior. On the other hand, outlets self-reported a prohibited behavior, administration of injections, at a similar level to client report which gives some reassurance as to the accuracy of the outlet reported data.

This study provides a robust comparison of thousands of clients served in retail outlets over the course of 24 months. However, there are limitations to consider. First, these comparisons are aggregated owing to the inability to link individual records in each dataset. We cannot rule out changes in outlet practice when dependent on the presence of the exit interviewers. However, we tried to mitigate this by conducting exit interviews on random days of the month, sending different interviewers in rotation, and interviewing clients away from the entrance of the outlet, ideally out of the line of sight of the outlet staff. A study conducted in health facilities showed no statistical evidence for change in provider behavior on interview days (24), giving us reasonable confidence that our results reflect true differences rather than temporary changes in behavior upon seeing an exit interviewer. However, there is the possibility for bias in reporting mRDT-tested clients over those without since mRDT testing was emphasized in the training and the app required them to use the camera function to document the test. In a third of the outlets, the parent study provided a modest payment (0.10USD) in association with mRDT testing, but did not find this to affect the testing rate (9) and is therefore not expected to have affected the results presented here. There were significant challenges in the use of the app more generally, including frequent turnover of outlet staff which necessitated retraining, frustrations with ease of use of the app, and poor network signal in some outlets, all of which likely diminished the quality or consistency of the app data. It is also important to keep in mind that both data sources excluded individuals who were not available at the shop for testing - almost 7.5% and 30.4% of clients, reported by the app and exit interviews respectively, were purchasing drugs on behalf of someone not present. An important strength of the study is that the reported client experience is independent of the outlet influence since clients were interviewed by neutral observers outside of the outlet.

## Conclusions

In Kenya, testing using mRDTs in private retail sector - where the majority of antimalarials are distributed – can fill an important gap in appropriate malaria case management. Nevertheless, contrasting outcomes reported by the providers and the clients highlight barriers to improving testing, adherence to malaria drugs, and challenges for monitoring case management in the retail sector. These barriers include ensuring accurate communication of results to the client, reluctance to withhold antimalarials from test-negative clients, and over-reporting of dispensing government-recommended antimalarials to test-positive clients. Very high use of antibiotics despite the test results, especially of malaria-positive clients, compounds the challenge of targeted fever treatment in the retail sector.

An appropriate provider behaviour change intervention package that considers how the use of mRDTs can be aligned with private provider business models may be more effective at promoting adherence to test results. Training to improve the communication and clinical counseling offered to clients should also be considered. Appropriate policy design for the local health service setting, which addresses health worker practice and client perceptions, should be put in place to ensure that there is rational use of both tests and treatments (25).

## Figures and Tables

**Figure 1:**
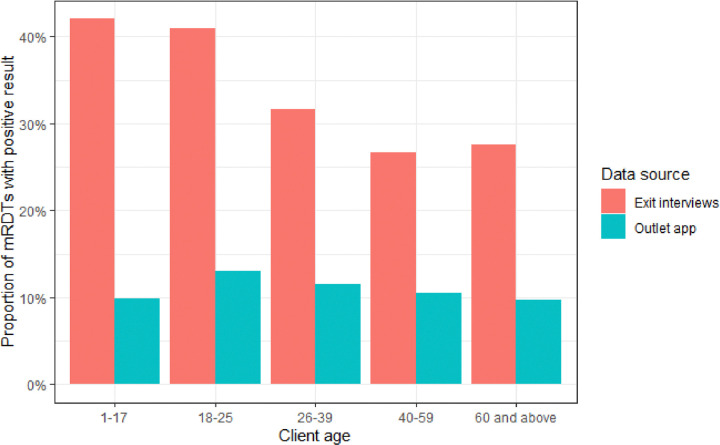
Positive mRDT by client age and data source

**Figure 2:**
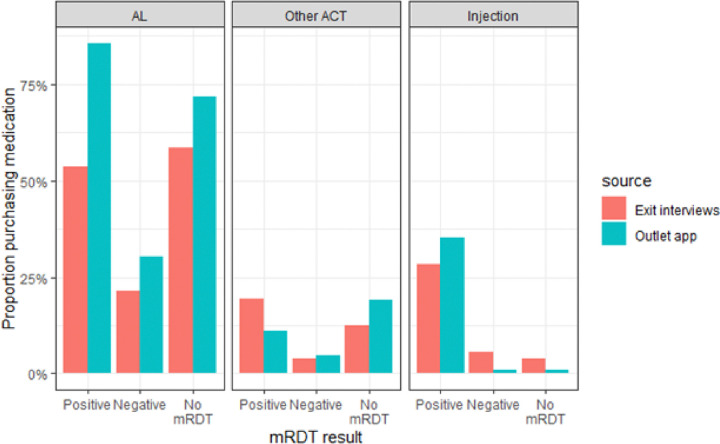
Medication purchase by test result and data source

**Table 1:** Summary of analysis population from the outlet’s mobile application (app) and the exit interviews

	Outlet app	Exit Interview
Number of observations	Encounters recorded*: 54,255	Clients screened*: 11,783
Ineligibility criteria**		
Client not present	4,095 (7.5%)	3,578 (30.4%)
Not known if client present	356 (0.7%)	NA
Already started antimalarials	NA	1,293 (11.0%)
Excluded other criteria	NA	960 (6.0%)
Not consented	NA	256 (2.2%)
Incomplete survey	NA	1 (<0.001%)
Eligible for current analysis (i.e. client present and no other exclusion criteria)	49,804 (91.8%)	5,695 (48.3%)
Gender of client (n, %) - Those included in the current analysis		
Male	24,639 (49.5%)	3,016 (53.0%)
Female	25,165 (50.5%)	2,679 (47.0%)
Age of client (n, %)- Those included in the current analysis		
1–17 years	17,510 (35.2%)	1,750 (30.7%)
18–25 years	9,950 (20.0%)	613 (10.8%)
26–39 years	13,499 (27.1%)	1,330 (23.3%)
40–59 years	7,375 (14.8%)	1,503 (26.4%)
60 years and above	1,470 (3.0%)	490 (8.6%)
Missing	0	9 (0.16%)
Tested at outlet by mRDT(n, %)		
Yes	25446 (51.1%)	2436 (42.8%)

* Note that these may not be unique individuals – an individual may have more than one encounter if they returned to the outlet with malaria-like symptoms during the study period.

** Note that multiple reasons may apply for ineligibility for inclusion in analysis of the exit interview data.

**Table 2:** Proportion of clients with a test at the outlet by gender and age

Variables	Male		Female	
	Outlet app N = 24,639	Exit Interview N = 3,016	Outlet app N = 25,165	Exit Interview N = 2,679
Proportion of clients tested at the outlet	12,096 (49.1%)	1,150 (38.1%)	13,350 (53.0%)	1,286 (48.0%)
Proportion of clients in each age category who were tested at the outlet				
1–17 yrs	4,602/8,662 (53.1%)	485/899 (54.0%)	4,765/8,848 (53.9%)	440/851 (51.7%)
18–25 yrs	2,217/4,613 (48.1%)	115/308 (37.3%)	2,872/5,337 (53.8%)	152/305 (49.8%)
26–39 yrs	2,895/6,678 (43.4%)	200/663 (30.2%)	3,388/6,821 (49.7%)	290/667 (43.5%)
40–59 years	1,917/3,910 (49.0%)	241/864 (27.9%)	1,898/3,465 (54.8%)	286/639 (44.8%)
60+ years	465/776 (59.9%)	109/276 (39.5%)	427/694 (61.5%)	116/214 (54.2%)
Missing	0	0	0	2/3 (66.7%)

**Table 3:** Test results by client age and gender Client characteristics by test result in the TESTsmART app and the exit interviews

		Outlet app (n = 25,407^2^)	Exit interviews (n = 2396^1^)
		mRDT Positive	mRDT Positive
Total		2,788 (11.0%)	842 (35.1%)
Gender	Male	1,322/12,096 (10.9%)	411/1131 (36.3%)
	Female	1,466/13,350 (11.0%)	431/1265 (34.1%)
Age	1–17 yrs	917/9,353 (9.8%)	383/911 (42.0%)
	18–25 yrs	664/5,080 (13.1%)	107/261 (41%)
	26–39 yrs	719/6,271 (11.5%)	152/480 (31.7%)
	40–59 years	401/3,811 (10.5%)	139/521(26.7%)
	60 + years	87/891 (9.8%)	61/221 (27.6%)

1 Excluding those who tested and did not know the result (n = 29) or had an invalid result (n = 9) or had missing age (n = 2)

2 Excluding those who tested and did not know the result or had an invalid result (n = 40)

**Table 4:** Number of different medications purchased and type by testing status

	Outlet app				Exit interview		
# medications purchased	No test (n = 24,358)	mRDT Pos (n = 2,788)	mRDT Neg (n = 22,619)	No test, client not present (n = 4,095)	No Test* (n = 3259)	mRDT pos (n = 842)	mRDT neg (n = 1554)
None	296 (1.2%)	24 (0.9%)	3,560 (15.7%)	131 (3.2%)	108 (3.3%)	55 (6.5%)	357 (23.0%)
1–2	17,334 (71.2%)	1,095 (39.3%)	13,287 (58.8%)	3,078 (75.2%)	2,545 (78.1%)	419 (49.8%)	782 (50.3%)
3–4	6,704 (27.5%)	1,484 (53.2%)	5,741 (25.4%)	883 (21.6%)	592(18.2%)	347 (41.2%)	402 (25.9%)
5 +	24 (0.1%)	185 (6.6%)	30 (0.1%)	3 (0.1%)	12 (0.4%)	21 (2.5%)	13 (0.8%)
Type of medication							
AL	17,509 (71.9%)	2,383 (85.5%)	6,872 (30.4%)	2,866 (70.0%)	1904 (58.4%)	451 (53.6%)	334 (21.5%)
Other ACT	4,679 (19.2%)	305 (10.9%)	1,065 (4.7%)	539 (13.2%)	404 (12.4%)	163 (19.4%)	59 (3.8%)
Other antimalarial	1,516 (6.2%)	83 (3.0%)	173 (0.8%)	530 (12.9%)	221 (6.8%)	22 (2.6%)	19 (1.2%)
Injection**	263 (1.1%)	983 (35.3%)	253 (1.1%)	0 (0.0%)	124 (3.8%)	239 (28.4%)	87 (5.6%)
Antibiotic	5,049 (20.7%)	1,176 (42.2%)	9,181 (40.6%)	530 (12.9%)	800 (24.6%)	375 (44.5%)	659 (42.4%)
Painkiller	17,725 (72.8%)	2,236 (80.2%)	14,420 (63.8%)	2,881 (70.4%)	2175 (66.7%)	664 (78.9%)	947 (60.9%)
Other	4,566 (18.8%)	868 (31.1%)	8,381 (37.1%)	625 (15.3%)	331 (10.2%)	136 (16.2%)	277 (17.8%)

* 9 clients reported invalid test results, and 29 did not know their test results. They are not included in this table.

** Injections were often reported with the name of the pathogen it was purported to treat (as in ‘malaria injection’ or ‘typhoid injection’), unknown injection, or just ‘injection’ and rarely ‘artemether injection.’ Most often, the specific drug was not captured because the client did not carry the packaging for the interviewer to copy the drug information. All injectable drug types are reported here. The ‘Other antimalarial’ category does not include injections.

**Table 5:** Exit testing

Test at outlet	Test at Exit interview		
	Negative	Positive	Total
Negative	20 (90.9%)	2 (9.1%)	22 (100%)
Positive	9 (64.3%)	5 (35.7%)	14 (100%)
Not tested	105 (96.3%)	4 (3.7%)	109 (100%)

## Data Availability

The datasets analysed during the current study are available from Duke University Libraries Digital Repository. https://research.repository.duke.edu/concern/datasets/x633f206c?locale=en.
